# Purification and characterization of detergent-compatible protease from *Aspergillus terreus* gr.

**DOI:** 10.1007/s13205-014-0200-6

**Published:** 2014-02-28

**Authors:** Francois N. Niyonzima, Sunil S. More

**Affiliations:** Department of Biochemistry, Center for Post Graduate Studies, Jain University, Bangalore, 560011 India

**Keywords:** Alkaline protease, *Aspergillus terreus* gr., Purification, Detergent

## Abstract

The possibility of using *Aspergillus terreus* protease in detergent formulations was investigated. Sodium dodecyl sulfate (SDS) and native polyacrylamide gel electrophoresis indicated that the purified alkaline protease (148.9 U/mg) is a monomeric enzyme with a molecular mass of 16 ± 1 kDa. This was confirmed by liquid chromatography–mass spectrometry. The active enzyme degraded the co-polymerized gelatin. The protease demonstrated excellent stability at pH range 8.0–12.0 with optimum at pH 11.0. It was almost 100 % stable at 50 °C for 24 h, enhanced by Ca^2+^ and Mg^2+^, but inhibited by Hg^2+^, and strongly inhibited by phenylmethyl sulfonyl fluoride. It showed maximum activity against casein followed by gelatin; its *V*_max_ was 12.8 U/ml with its corresponding *K*_M_ of 5.4 mg/ml. The proteolytic activity was activated by Tween-80, Triton-100 and SDS, and remained unaltered in the presence of H_2_O_2_ and NaClO. The enzyme exhibited higher storage stability at 4, 28 and −20 °C. It was stable and compatible to the desired level in the local detergents. The addition of the protease to the Super wheel improved its blood stain removal. The isolated protease can thus be a choice option in detergent industry.

## Introduction

Proteases (EC 3.4.21–24 and 99) are hydrolytic enzymes that cleave peptide bonds of proteins. The extracellular proteases of microbial origin are important enzymes and account for approximately 60 % of the total worldwide enzyme sale (Rao et al. 1998). Alkaline proteases are primarily used in the detergent industry and need not to be in pure form. However, proteases that are used in other areas such as pharmaceutical and medical applications require high purity (Kumar and Takagi 1999). Choudhary and Jain (2012) reported that enzyme purification is tedious, time consuming and very expensive. The precipitation is the most common method used to partially purify and concentrate the protein from fermentation crude extract (Bell et al. 1983). It is performed by the addition of inorganic salt like ammonium sulfate or organic solvent such as acetone or ethanol (Kumar and Takagi 1999). Alkaline proteases generally do not bind to anion exchangers as they are generally positively charged. The cation exchangers can thus be used and the elution of the bound molecules can be done by increasing the salt or pH gradient (Fujiwara et al. 1993). The lectin-agarose affinity chromatography is also used to separate glycoproteins from non-glycosylated proteins (Kobayashi et al. 1996).

The study of alkaline protease properties is important from the point of view of its practical applicability. Alkaline proteases used in detergent preparations must have higher activity at alkaline pH, broad temperature range, broad substrate specificity, stability in the presence of surfactants, oxidizing agents, and compatibility with detergents (Kumar and Takagi 1999; Adinarayana et al. 2003; Choudhary and Jain 2012). The cations like Ca^2+^, Mg^2+^ and Mn^2+^ usually increased the activity and stabilized the enzyme (Sharma et al. 2006; Anandan et al. 2007; Kalpana devi et al. 2008; Dubey et al. 2010). The nature of the enzyme and its active site as well as its cofactor requirements can be deduced from inhibition studies (Sigma and Mooser 1975). Alkaline proteases are differently affected by various inhibitors. However, most of the fungal alkaline proteases are generally serine proteases (Coral et al. 2003). The alkaline proteases have in general the broad substrate specificity and breakdown a variety of natural substrates and synthetic substrates (Kumar and Takagi 1999).

Enzymes are used in a very small amount in detergent preparations to increase the cleaning ability of detergents (Bajpai and Tyagi 2007). If a detergent does not contain an enzyme, it may not completely remove the stains resulting in permanent residues (Hasan et al. 2010). The performance of an enzyme in a detergent is based on the detergent composition, type of stains to be removed, water hardness, washing temperature and procedure (Hasan et al. 2010). Kirk et al. (2002) emphasized that the detergent protease must work at room temperature to save energy. The cleansing process is the reverse process of coagulation and adhesion and requires energy from external sources such as human hands and washing machine. In the presence of a detergent, the energy gets reduced (Bajpai and Tyagi 2007).

The industrial demand of proteases with novel and better properties continues to stimulate the researchers in this area. For the production of proteases for industrial use, isolation, purification and characterization of new promising strain are continuous processes (Kumar et al. 2002). Although different alkaline proteases have been isolated from several bacteria and fungi, few have better properties that can be commercially exploited. *Aspergillus terreus* gr. was recently identified as a potent producer of an extracellular stable alkaline protease (Niyonzima and More 2013). The aim of the present study was, therefore, to purify and characterize an alkaline protease of *A. terreus* as well as to ascertain its suitability as detergent additive.

## Materials and methods

### Fungal strain and fermentation conditions

The alkaline protease-producing fungus used was isolated from potato grown soil fields of Bangalore and identified as *A. terreus* gr. based on morphological and microscopic features and it has been deposited into the National Fungal Culture Collection of India (Agarkar Research Institute, Pune). The composition of the fermentation medium found after optimization consisted of (w/v) 1.5 % casein, 0.1 % KH_2_PO_4_, 0.1 % K_2_HPO_4_, 0.02 % MgSO_4_ and 2 % soybean meal, pH 10.0. The optimized fermentation medium was inoculated with 2 % (v/v) inoculum (2 × 10^6^ spores/ml) and incubated at 37 °C for 5 days. After incubation, the culture broth was centrifuged (REMI C-30 BL Cooling centrifuge, India) at 10,000 rpm for 10 min at 4 °C. The supernatant was used as a crude enzyme (Niyonzima and More 2013).

### Alkaline protease activity and protein estimation

The proteolytic activity was determined as per Niyonzima and More (2013) using casein as the substrate. Protein concentration was estimated as per Lowry et al. (1951).

### Purification of alkaline protease

The method of Kim et al. (1996) with slight modifications was followed to partially purify the alkaline protease of *A. terreus* gr. To one volume of supernatant, 3 volumes of cold acetone (−20 °C) was slowly added. The acetone precipitate was then collected by centrifugation at 13,000 rpm for 10 min after an incubation period of 3 h at −20 °C. A minimum volume of 0.1 M Tris–HCl buffer (pH 9.0) was used to dissolve the precipitate (0.5 ml for 100 mg). The resulted enzyme solution was subjected to lyophilization (Freeze dryer, Model LY3TTE, Snijders Scientific, Tilburg Holland) and the resulted powder served as the partially purified alkaline protease. Affinity chromatography method using agarose-bound lectins (Spivak et al. 1977) was used with minor modifications to completely purify the protease. The lectin concanavalin A (Con A) was chosen as the ligand. A 0.5 × 9 cm Con A-agarose column (2-ml bed volume) was prepared (10 mg/ml) and equilibrated with 0.1 M Tris–HCl buffer (pH 9.0). The lyophilized sample collected in the above step was dissolved in the same buffer and loaded into the column. Fractions were collected for the bound and unbound proteins and subjected to the lyophilization. The bound protein was eluted with buffer containing sucrose of 1 M strength. The protein content and enzyme activity were determined for both samples as described earlier. The adsorbed protein showing higher activity was used as the purified alkaline protease.

### SDS-PAGE

Sodium dodecyl sulfate- and native polyacrylamide gel electrophoresis (SDS-PAGE) was performed under non-reducing conditions (Laemmli 1970). Electrophoresis was carried out at 50 V in the stacking gel (6 %) and at 100 V in the separating gel (15 %). The gel was stained (0.25 % coomassie brilliant blue R 250, 15 % methanol, 7.5 % glacial acetic acid) for 2 h and destained overnight with stain solution excluding the dye. The protease was sized with the help of the protein molecular weight marker.

### Native PAGE

The purity and the nature of the protease were studied as per Laemmli (1970) using 15 % native PAGE. The procedure used was the same as described for SDS-PAGE except that the SDS was not included in the gel and electrophoretic solutions, and the samples were not heated.

### Gelatin zymography

The zymography was carried out in 15 % polyacrylamide gel containing 0.1 % (w/v) gelatin as a co-polymerized substrate (Heussen and Dowdle 1980). The native PAGE band was extracted and mixed with the sample buffer without heat denaturation prior to electrophoresis. Electrophoresis was carried out as described for SDS-PAGE. After electrophoresis, the gel was placed in a tray on a gel rocker and washed twice with a wash buffer (2.5 % Triton X-100 in distilled H_2_O, 0.02 % NaN_3_) for 20 min each to remove the SDS. It was then rinsed for 10 min in a 50 ml incubation buffer (50 mM Tris–HCl, pH 8.0, 5 mM CaCl_2_, 0.02 % NaN_3_). The gel was placed in 150 ml fresh incubation buffer and incubated at 37 °C for 24 h to allow the gelatin degradation. The staining and destaining were the same as described for SDS-PAGE. The activity band was indicated by a clear, colorless zone against the blue background.

### LC–MS

LC–MS of the purified protein was carried using an Agilent 1290 Infinity LC system coupled to an Agilent 6530 Q TOF with an Agilent Poroshell C18, 75 × 2.1 mm, 5 μm analytical column. 0.1 % formic acid in water (A) and 90 % acetonitrile in water with 0.1 % formic acid (B) served as solvents for LC. Before MS analysis, 3 % acetonitrile/water with 0.1 % formic acid solution was utilized to dilute the protein samples. The LC gradient started with 3 % B and in 15 min it went up to 95 % B and then returned back to 3 % B by 16 min. Spectra were noted in positive ion and in profile mode. Data were acquired on standard (3,200 *m*/*z*), 2 GHz, MS only mode, range 500–3,200 *m*/*z*. Agilent Mass Hunter Qualitative Analysis software was used to analyze the data. The protein masses were obtained by deconvoluting mass spectrum using the maximum entropy algorithm in Bioconfirm module. The molecular mass was determined by a minimum of five peaks well above the baseline.

### Effect of pH on activity and stability of the alkaline protease

The effect of pH on alkaline protease activity was studied by pre-incubating 0.05 ml of enzyme in 0.95 ml of the concerned buffer at room temperature (28 ± 2 °C) for 30 min. After pre-incubation, the assay was continued as reported earlier. pH 10.0, 11.0 and 12.0 were used to study the stability of the enzyme. For each pH, 5.7 ml of a given buffer was mixed with 0.3 ml of the enzyme and mixed well. The enzyme reaction was followed at room temperature and the residual activity was noted after 0, 30 min, 1, 2, 4 and 24 h by considering maximum activity as 100 %.

### Effect of temperature on activity and stability of the alkaline protease

The mixture of 0.05 ml of alkaline protease and 0.95 ml of 0.1 M glycine–NaOH buffer (pH 11.0) was treated at different temperatures ranging from 0 to 90 °C for 30 min. The enzyme activity was determined as above. Thermal stability was examined by incubating 0.3 ml of the enzyme with 5.7 ml of 0.1 M glycine–NaOH buffer (pH 11.0) at 50, 60, 70 and 80 °C. The residual activity was recorded after 0, 30 min, 1, 2, 4 and 24 h and was expressed as percentage of initial activity taken as 100 %.

### Effect of various metal ions on alkaline protease activity

0.05 ml of alkaline protease was mixed with 0.95 ml of 10 mM of the chloride under study (chloride of Ca, Mg, Co, Cu, Fe, Hg, K, Mn, Zn, Co and Na) and incubated for 30 min at 50 °C. The residual alkaline protease activity was assessed by the standard assay procedure and was expressed as percentage of activity without metal ion, considered as 100 %.

### Effect of different group-specific reagents on the activity of alkaline protease

The specific reagents evaluated were EDTA, urea, sodium azide (NaN_3_), tosyl-l-lysylchloromethyl ketone (TLCK), dithiothreitol (DTT), iodoacetamide (IAA), phenylmethyl sulfonyl fluoride (PMSF),*N*-ethylmaleimide (NEM), *N*-Diazoacetylnorleucine methyl ester (DAN) and *N*-bromosuccinimide (NBS). The purified alkaline protease (0.05 ml) was mixed with 0.95 ml of 5 mM specific inhibitor and pre-incubated for 30 min at 50 °C. The residual activity was assessed using control which was without inhibitor and was taken as 100 %.

### Broad substrate specificity

Casein, gelatin, BSA, egg albumin and fibrinogen were used to study the substrate specificity of the alkaline protease. 0.95 ml of 0.1 M glycine–NaOH buffer (pH 11.0) was mixed with 0.05 ml of the purified enzyme. The mixture was incubated at 50 °C in the presence of 2 ml of 0.65 % (w/v) substrate for 10 min. The activity was determined as earlier.

### Determination of kinetic parameters

The various casein concentrations (0–20 mg/ml) in 0.1 M glycine–NaOH buffer (pH 11.0) were incubated for 10 min with equal amounts of the alkaline protease (0.1 ml of 1 mg/ml) at 50 °C. The enzyme activity was then recorded as earlier. The *K*_M_ and the maximum rate of reaction (*V*_max_) were evaluated graphically (Lineweaver and Burk 1934).

### Compatibility of alkaline protease with detergent components

The enzyme was tested for its stability in the presence of detergent components. The surfactants used were SDS, Triton X-100 and Tween-80, whereas the oxidizing and bleaching agents were H_2_O_2_ and NaClO, respectively (Pathak and Deshmukh 2012). 0.95 ml of 1 % (and 5 %) surfactant/oxidizing agent was mixed with 0.05 ml of enzyme in 0.1 M glycine–NaOH buffer (pH 11.0) and pre-incubated for 30 min at 50 °C. The residual activity of protease was assessed as earlier. The control was without any additive.

### Storage stability of alkaline protease

The storage stability of the enzyme at −20, 4 and 28 °C was analyzed. The enzyme preparation (30 mg/ml) was stored at the corresponding temperature and the residual activity was recorded by the standard assay procedure as described previously after exactly 10 days for a period of 40 days. The relative activity at day one was considered as 100 % (Beena et al. 2012).

### Compatibility of alkaline protease with local detergents

Ariel (Procter and Gamble Home Products Ltd-Mumbai, India), Henko (Henkel Spic, India), More choice (San Soaps and Detergents, Bengaluru, India), Super wheel and Surf excel (Hindustan Unilever Limited-Mumbai, India) were used as local detergents. They were diluted in tap water to a final concentration of 0.7 % (w/v) and heated at 95 °C for 10 min (Adinarayana et al. 2003; Kalpana devi et al. 2008; Dubey et al. 2010). A reaction mixture comprising 0.3 ml of enzyme and 5.7 ml detergent was pre-incubated at room temperature (28 ± 2 °C). The residual activity was recorded at 0, 30 min, 1, 2, 4 and 24 h. The procedure was repeated at 60 and 90 °C. The relative activity was expressed as percentage enzyme activity taking the activity of the control sample without a detergent as 100 % (Ali 2008).

### Cleansing potential of the alkaline protease as a detergent additive

The small white cloth pieces (4 × 4 cm) were stained with blood and air dried for a week. The stained clothes were incubated in 50 ml tap water + 1 ml of 7 mg/ml detergent, and in a mixture of 50 ml tap water, 1 ml detergent solution (0.7 %, w/v) and 1 ml of alkaline enzyme (20 mg/ml). The visual examination of air dried cloth pieces after clean tap water rinse was used to assess the stain removal. The unused stained cloth was used as the control (Adinarayana et al. 2003).

### Statistical analysis

Three independent experiments for each treatment were performed to determine enzyme activity. The means were compared by ANOVA and means for groups in homogeneous subsets were given by Duncan’s multiple range test (DMRT) at the 5 % significance level. The SPSS statistical package (PASW Statistics 18) was used for statistical evaluations.

## Results and discussion

### Purification of alkaline protease of *Aspergillus terreus* gr.

The fungus was grown under submerged fermentation since it was found to be appropriate and simple method of producing in bulk the extracellular alkaline protease (Niyonzima and More 2013). The enzyme was partially purified by acetone precipitation from crude enzyme and further totally purified by the affinity chromatography with agarose-bound lectins. A 28.6 purification fold with a yield in enzyme activity of 38.5 % and a specific activity of 148.9 U/mg was recorded. The purification results are summarized in Table 1. Anandan et al. (2007) reported 21-purification fold for an alkaline protease of *Aspergillus tamarii* with 905.7 U/mg and 8 % yield. Charles et al. (2008) recovered 58 % alkaline protease of *Aspergillus nidulans* HA-10 with a specific activity of 424 U/mg. A low recovery of 3.2 % alkaline protease of *Aspergillus flavus* with 170 U/mg and 5.8-purification fold was noted by Muthulakshmi et al. (2011). A better yield was also recorded for the alkaline protease when acetone was used as a primary precipitating agent (Kim et al. 1996). Concanavalin A affinity chromatography also gave a significant yield for the protease purification from *Clitocybene bularis* (Sabotiè et al. 2009).

**Table 1:** Summary of purification of alkaline protease of *A. terreus* gr.

Fraction	Total activity (U)	Total protein (mg)	Specific activity (U/mg)	Purification fold	Yield of activity (%)
Cell-free extract	240	46	5.2	1	100
Acetone precipitate	174	12.8	13.6	2.6	72.5
Purified enzyme by affinity chromatography	92.3	0.62	148.9	28.6	38.5

### SDS-PAGE and molecular weight determination

**Fig. 1:**
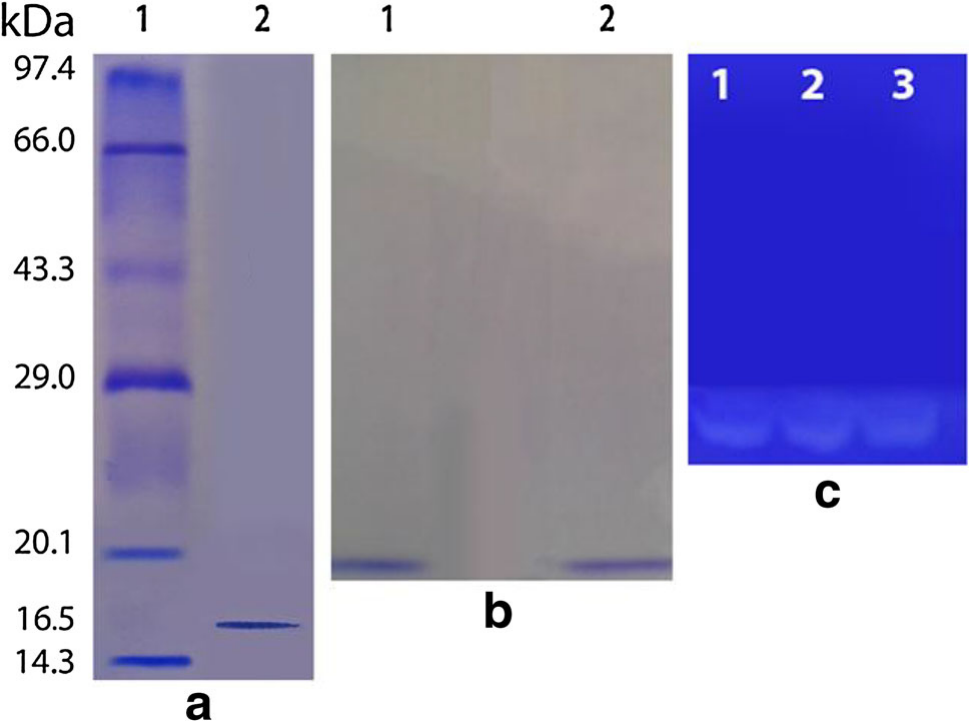
**a** SDS-PAGE of the purified protease: *Lane 1* protein molecular weight marker, *Lane 2* purified alkaline protease. **b** Native PAGE of purified alkaline protease: *lane 1* and *2*. **c** Activity staining of the native PAGE gel: gelatin degradation is shown by a clear colorless zone against the *blue* background

**Fig. 2:**
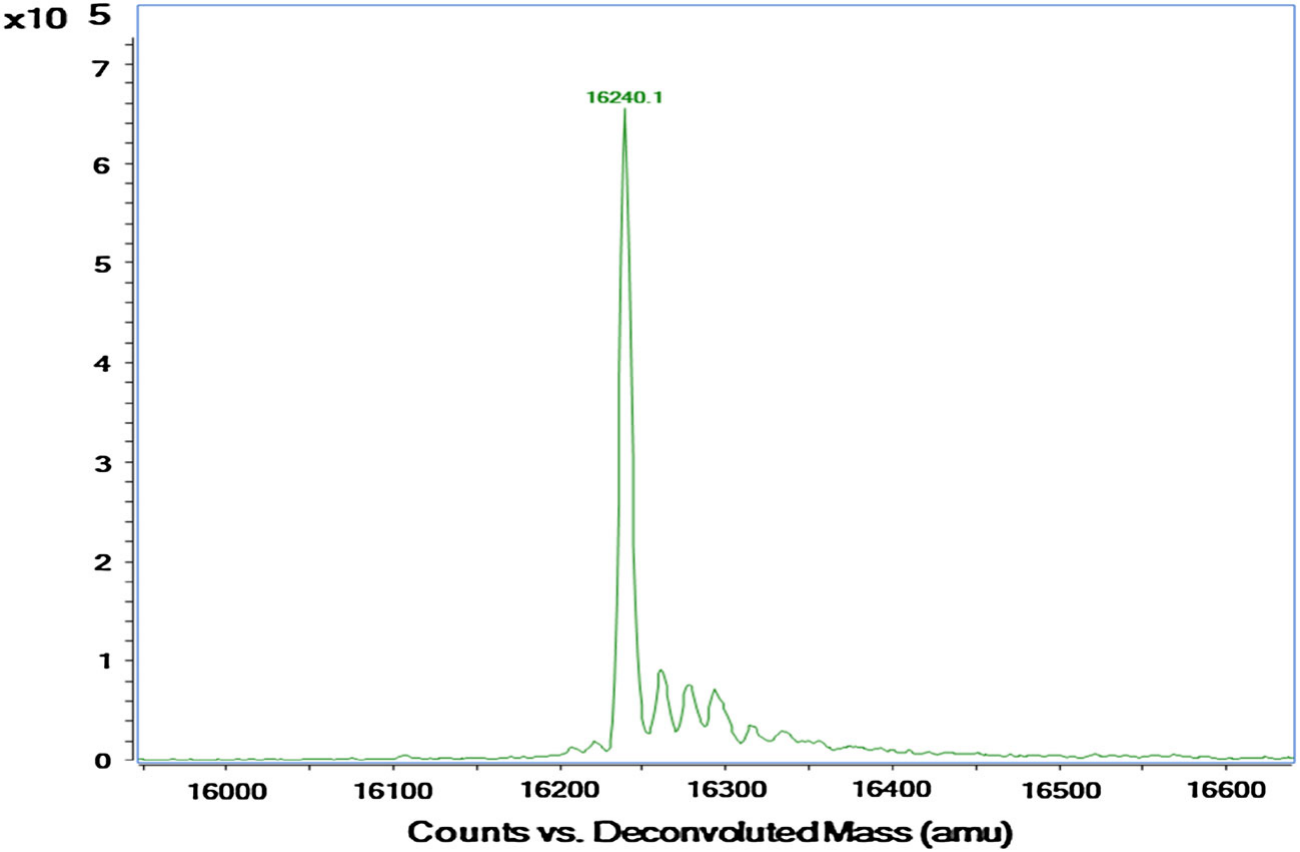
Deconvoluted mass spectrum of alkaline protease of *A. terreus* gr.

The molecular weight of the protein band was 16 ± 1 kDa. The purified enzyme was homogenous showing a single band (Fig. 1a). SDS-PAGE is the most widely used method to monitor protein purification and to size proteins. However, the determination of the relative molecular weight must be confirmed by another method like mass spectrometry. Figure 2 shows the deconvoluted mass spectrum of the alkaline protease of *A. terreus* as analyzed by the LC–MS. The protein showed a mass of 16.24 kDa. This also correlated with the SDS-PAGE analysis. A low molecular weight of 15 kDa was also reported for bacterial alkaline protease (Adinarayana et al. 2003). From the viewed reports, the molecular weight of *Aspergillus* proteases ranges from 23 to 124 kDa. For examples, 124 kDa was recorded for serine protease from *Aspergillus fumigatus* by Wang et al. (2005), 68 kDa from *Aspergillus niger* Z1 (Coral et al. 2003), 45 kDa from *A. tamarii* (Anandan et al. 2007), 33 kDa from *Aspergillus oryzae* AWT 20 (Sharma et al. 2006), and 23 kDa from *Aspergillus parasiticus* (Tunga et al. 2003). The molecular weights of alkaline proteases of *Aspergillus* species are thus different from one another which may be ascribed to genetic differences.

### Native PAGE and gelatin zymography

A single band at 16 ± 1 kDa was also seen (Fig. 1). The appearance of a single band for both native and SDS (Fig. 1a, b) gels suggested the purified alkaline protease to be a pure monomeric enzyme. To be convinced that the native PAGE band was not a contaminant; the gelatin zymography was carried out to show the activity of alkaline protease band. A clear colorless zone (of gelatin hydrolysis) against the blue background was observed (Fig. 1c). This further confirmed the homogeneity observed in both native and SDS-PAGE gels. The colorless zone resulted from the substrate degradation by proteases of *Aspergillus* species was also reported (Anandan et al. 2007; Charles et al. 2008).

### Effect of pH on activity and stability of the alkaline protease

The enzyme was inactive at low pH and active over a broad alkaline pH range (8.0–12.0) with maximum activity at pH 11.0 (Table 2). A high protease activity in the alkaline region can be attributed to the better binding of the enzyme to the substrate since the pH strongly decides the binding of the enzyme to the substrate (Takami et al. 1990). The optimum pH in the 7.5–10.0 was reported for most of the alkaline proteases of *Aspergillus* species (Coral et al. 2003; Hossain et al. 2006; Sharma et al. 2006; Anandan et al. 2007; Ali 2008; Charles et al. 2008; Dubey et al. 2010; Choudhary and Jain 2012). The pH 10.5 was also observed for important enzymes used in detergents (subtilisin Carlberg and subtilisin Novo or BPN17) (Adinarayana et al. 2003). The variation in alkaline protease activities at different pH optima may be ascribed to the genetic variability among the different *Aspergillus* species.

**Table 2:** Effect of pH, temperature, cations and specific inhibitors on the alkaline protease activity

pH	Enzyme activity (U/ml)	Temp. (°C)	Enzyme activity (U/ml)	Cation (10 mM)	Residual activity (%)	Specific inhibitor (5 mM)	Residual activity (%)
2	2.5 ± 0.5^e^	0	40.1 ± 2.7^b^	Control	100.0 ± 0.0^c,d^	Control	100.0 ± 0.0^a^
3	4.5 ± 0.6^e^	10	40.4 ± 1.3^b^	Mn^2+^	85.9 ± 14.8^d,e^	PMSF	16.3 ± 3.7^e^
4	6.3 ± 1.5^e^	20	42.6 ± 2.3^b^	Fe^3+^	81.1 ± 9.7^d,e^	TLCK	93.3 ± 3.9^a,b^
5	13.5 ± 0.6^d^	30	43.2 ± 0.2^b^	Hg^2+^	74.8 ± 9.6^e^	DTT	90.1 ± 2.4^a,b^
6	28.7 ± 5.0^c^	40	45.2 ± 1.6^b^	Mg^2+^	152.2 ± 17.6^b^	IAA	94.8 ± 2.1^a,b^
7	33.3 ± 1.1^b^	50	52.8 ± 2.5^a^	Ca^2+^	182.6 ± 9.9^a^	NBS	49.7 ± 9.7^d^
8	37.3 ± 0.7^a,b^	60	52.3 ± 0.3^a^	Co^2+^	121.1 ± 9.8^c^	NEM	88.8 ± 9.7^a,b^
9	38.7 ± 3.5^a^	70	52.1 ± 1.8^a^	Zn^2+^	89.1 ± 12.8^d,e^	NaN_3_	78.0 ± 11.0^c^
10	40.0 ± 0.9^a^	80	51.8 ± 0.8^a^	Cu^2+^	100.67 ± 1.2^c,d^	EDTA	87.3 ± 5.7^b,c^
11	40.9 ± 3.0^a^	90	35.5 ± 2.3^c^	Fe^2+^	99.7 ± 8.1^c,d^	DAN	91.7 ± 5.3^a,b^
12	40.6 ± 2.1^a^			Ba^2+^	102.88 ± 11.0^c,d^	Urea	95.5 ± 4.1^a,b^
13	24.9 ± 4.0^c^			K^+^	97.4 ± 4.4^d^		
				Na^+^	92.54 ± 13.2^d^		

The values bearing the same letters in a column do not differ significantly at *P*_0.05_

The stability of the protease was studied at three different pHs viz. pH 10.0, 11.0 and 12.0. The enzyme remained unaltered at pH 11.0 and 12.0 for 24 h at 28 °C and a slight decrease in relative activity was seen at pH 10.0 after 24 h (data not shown). Good stability was also seen for an alkaline protease of *Aspergillus* species in the range of pH 8.0–9.0 for 20 min at 40 °C (Choudhary and Jain 2012), 7.0–11.0 at 50 °C for 1 h (Kalpana devi et al. 2008) and 6.0–12.0 for 30 min at 30 °C (Dubey et al. 2010). The enzyme recovered from the present study appeared to have higher stability than others purified from *Aspergillus* species since no one retained a significant activity after 24 h, suggesting the enzyme to be used in detergent preparations.

### Effect of temperature on activity and stability of the alkaline protease

The enzyme showed higher activity for most of the temperatures tested with an optimum at 50 °C although statistically at par with 60, 70 and 80 °C (Table 2). The appreciable activity of the alkaline protease at low, ambient and higher temperatures highlights its capacity to be used at any washing temperature. Beena et al. (2012) also purified the protease that was capable of being used in cold and hot washing conditions. The maximum activity at 50 °C was also recorded, while working with the protease of *Aspergillus* species (Kalpana devi et al. 2008; Muthulakshmi et al. 2011). Most of the optimum temperatures reported for alkaline proteases of *Aspergillus* species are in the 30–45 °C range (Coral et al. 2003; Hossain et al. 2006; Anandan et al. 2007; Ali 2008; Charles et al. 2008; Dubey et al. 2010; Choudhary and Jain 2012).

When the thermostability profile examined, the retention of full activity was seen for over 4, 2, 1 h and 30 min at 50, 60, 70 and 80 °C, respectively. However, a loss in relative activity in the range of 1.3 % (50 °C) to 19.6 % (at 80 °C) was noted after 24 h (data not shown). The alkaline protease secreted by *A. terreus* retained full activity in the 40–90 °C range, but only for 1 h (Ali 2008). The alkaline protease produced by *A. niger* was 100 % stable at 40 °C for 1 h (Kalpana devi et al. 2008). The enzyme of the present study had a higher stability demonstrating its suitability in detergent formulations. This thermostability can be attributed to its glycoproteinic nature. An increased thermostability of many glycosylated proteins at elevated temperatures was due to the presence of the carbohydrate moiety (Ahmed et al. 2007).

### Effect of various metal ions on alkaline protease activity

Ca^2+^ and Mg^2+^ enhanced the alkaline protease activity, while Co^2+^, Fe^2+^, Ba^2+^, K^+^, Na^+^ and Cu^2+^ did not show any alteration in enzyme activity. A slight reduction in alkaline protease activity was observed with Mn^2+^, Fe^3+^ and Zn^2+^; however, a loss in enzyme activity of 25 % was observed with Hg^2+^ (Table 2). The stimulation of alkaline protease of *Aspergillus* species by Ca^2+^ and Mg^2+^ has been previously described (Sharma et al. 2006; Anandan et al. 2007; Kalpana devi et al. 2008; Dubey et al. 2010) suggesting the vital role of these cations in maintaining the active site and thus improving the enzyme thermostability. Similar to the alkaline protease of the present study, Mn^2+^, Fe^2+^ and Zn^2+^ also inhibited the alkaline protease purified from *Aspergillus* species (Anandan et al. 2007; Kalpana devi et al. 2008). The enzyme was labile in the presence of Hg^2+^ as 25 % of the activity was lost. Hg^2+^ was also inhibited by the alkaline proteases of *Aspergillus* species (Sharma et al. 2006; Anandan et al. 2007).

### Effect of different group-specific reagents on the activity of alkaline protease

The alkaline protease was unaltered by TLCK, DTT, IAA, NEM, EDTA, urea and DAN. A slight, moderate and strong inhibition was observed with NaN_3_, NBS and PMSF, respectively (Table 2). The strong inhibition of the protease by PMSF suggested the serine residue in the active site. The essential serine residue was reported for the alkaline proteases of *Aspergillus* species (Coral et al. 2003; Hossain et al. 2006; Sharma et al. 2006; Anandan et al. 2007; Charles et al. 2008; Dubey et al. 2010). The moderate inhibition of alkaline protease by NBS may indicate that the tryptophan is near to the active site. Since the alkaline protease retained full activity in presence of TLCK, DTT, IAA, NEM, urea and DAN, the histidine and cysteine residues as well as sulfhydryl groups are not involved in the alkaline protease catalysis. Similarly, the –SH group was not involved in the alkaline protease of *A. tamarii* (Anandan et al. 2007). In this work, the enzyme activity was not lost in the presence of EDTA. Lack of inhibition by EDTA suggested that the enzyme not to be a metalloprotein. A similar result has been reported for alkaline proteases recovered from a fungal crude extract (Sharma et al. 2006; Anandan et al. 2007). The stability of protease in the presence of chelating agents like EDTA is a requirement for any detergent enzyme since EDTA is used in detergent formulation as a water softener (Beg and Gupta 2003).

### Broad substrate specificity

The enzyme was able to hydrolyze all the tested substrates with the greatest activity against casein although statistically at par with gelatin (data not shown). Similarly, the alkaline protease of *Aspergillus tamarii* was active towards casein than hemoglobin (Anandan et al. 2007). The alkaline protease of *A. niger* was also more active against casein than BSA and gelatin (Dubey et al. 2010). In contrast, the alkaline protease of *A. flavus* was active against gelatin than casein, egg albumin, and the BSA (Hossain et al. 2006). Since the alkaline protease of *A. terreus* gr. has the ability to hydrolyze various protein substrates, it can be a candidate of choice in detergent formulations as protein stain removal.

### Determination of kinetic parameters

The *K*_M_ and *V*_max_ of the purified alkaline protease were determined from Lineweaver–Burk plot as 5.4 mg/ml and 12.8 U/ml, respectively (data not shown). Similarly, the *K*_M_ of 5 mg/ml was calculated for the alkaline protease purified from *A. oryzae* AWT 20 (Sharma et al. 2006). 0.6 mg/ml and 60 U/mg were recorded for the alkaline protease of *A. flavus* (Muthulakshmi et al. 2011), while 0.8 mg/ml and 85 U/mg of protein were seen for the alkaline protease of *A. niger* (Kalpana devi et al. 2008). The lower *K*_M_ seen indicated the higher affinity of the protease of *Aspergillus* species towards their substrates.

### Compatibility of alkaline protease with detergent components

**Fig. 3:**
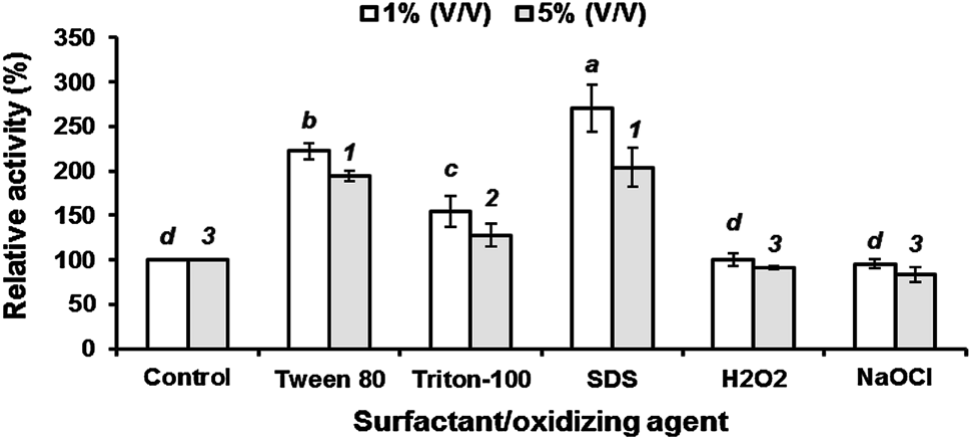
Stability of the alkaline protease in various detergent components. The values bearing the same letters or numbers do not differ significantly at *P*_0.05_

The stability of the enzyme in the presence of NaClO, H_2_O_2_, Tween 80, Triton X-100 and SDS was analyzed. The surfactants increased the activity (Fig. 3). The activation of alkaline protease in the presence of SDS indicates its suitability as detergent constituent. The mechanism proposed by Bajpai and Tyagi (2007) is that the negative charges of the SDS react with positive charges of calcium and magnesium present in the hard water/wash water thereby deactivating them. A better performance of protease was also observed with nonionic surfactant (Tween 80) (Beena et al. 2012). The enzyme was 100 % stable in the presence of bleaching and oxidizing agents (Fig. 3). It can thus withstand bleaching and oxidizing reactions taking place during washing. As the concentration of the concerned compound increased from 1 to 5 %, a slight to moderate decrease in enzyme activity was noted (Fig. 3). Low concentration of surfactants and oxidizing agents is thus necessary for alkaline protease-based detergent formulations.

### Storage stability of alkaline protease

The alkaline protease was stable for over the whole storage period studied as only 4, 7 and 9 % of its initial activity was lost after 40 days at 4, 28 and −20 °C, respectively (data not shown). The protease inhibitors would have contributed to the marginal decline in enzyme activities on prolonged storage conditions. Low value was observed at −20 °C which may be due to ice crystal formation that inactivates enzymes (Beena et al. 2012). The long shelf life of the protease suggested its utilization in detergent industry.

### Compatibility of alkaline protease with local detergents

**Fig. 4:**
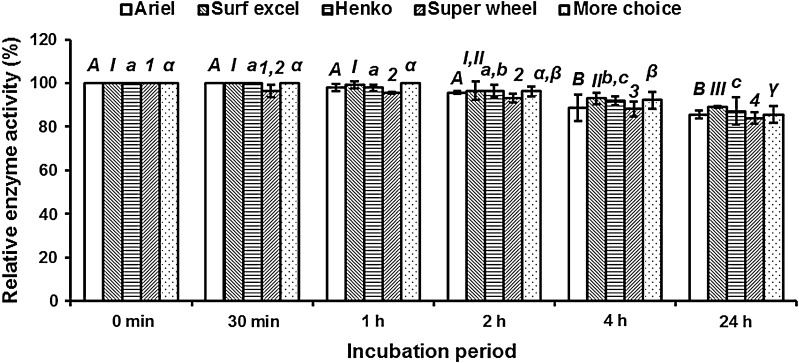
Stability and compatibility of enzyme with detergents. The values bearing the same letters or numbers for each detergent do not differ significantly at *P*_0.05_

To check if the enzyme can be commercially exploited in detergent industry, its stability and compatibility with 5 local powder detergents were investigated at room temperature (28 ± 2 °C), 60 and 90 °C. The protease was 100 % stable and compatible for 2 h at 60 °C with all the detergents except for Super wheel, and a retention of 83.98, 85.50, 85.57, 87.14 and 89.16 % was recorded after 24 h for Super wheel, More choice, Ariel, Henko and Surf excel, respectively (Fig. 4). The alkaline protease was also active and retained 79.06–83.2 and 55.75–75.14 % with tested detergents at 28 and 90 °C, respectively, after 24 h (data not shown).The detergent compatibility of the alkaline protease of *Aspergillus* species has been reported (Ali 2008; Kalpana devi et al. 2008; Dubey et al. 2010; Choudhary and Jain 2012), but no one was able to retain maximum activity after 1 h. The difference in enzyme stability of *Aspergillus* species in the presence of local detergents may be ascribed to the detergent composition. The enzyme was more active at room temperature and at 60 °C than at 90 °C. The high stability seen at room temperature had a good impact in saving electricity and in maintaining the quality of clothes.

### Cleansing potential of the alkaline protease as a detergent additive

**Fig. 5:**
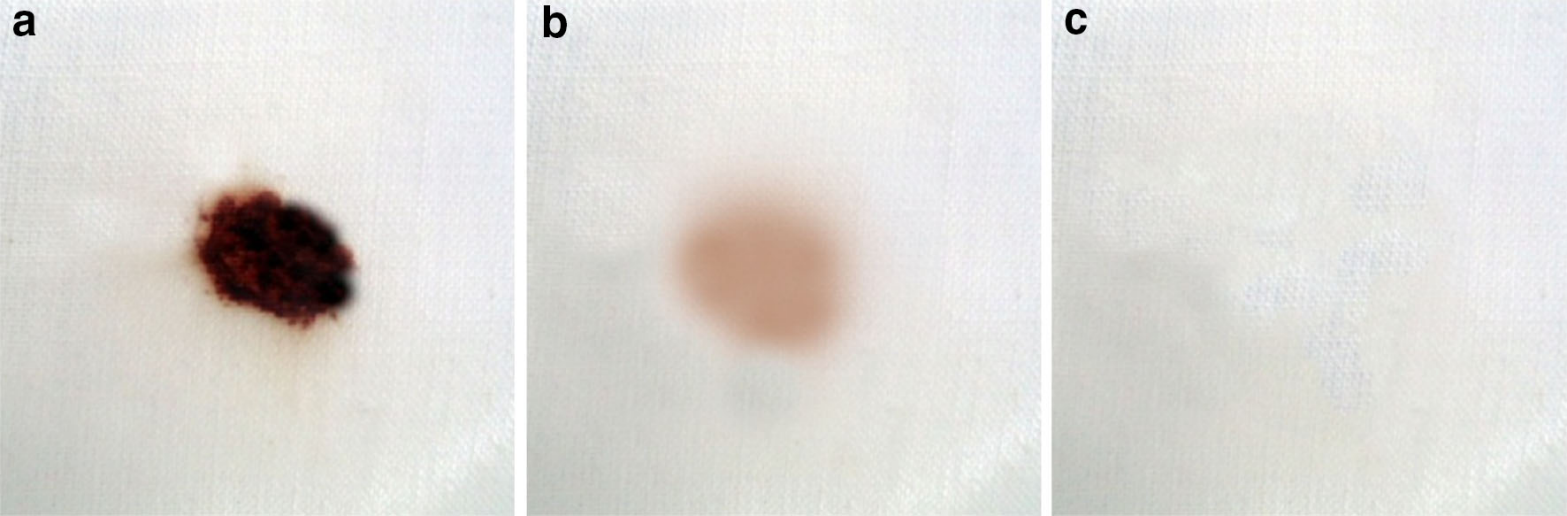
Blood stain removal analysis of enzyme preparation. Unwashed stained cloth (**a**), blood stained cotton cloth washed with Super wheel and tap water (**b**), blood stained cloth washed with alkaline protease, super wheel and tap water (**c**)

The protease-Super wheel (Non enzymatic commercial detergent) preparation was able to completely remove blood stain from white cotton fabric (Fig. 5). Similarly, the blood stain removal was significantly improved by the alkaline protease supplementation to a detergent (Adinarayana et al. 2003; Kalpana devi et al. 2008). As the blood stained was removed at a lesser time when a detergent solution was with an enzyme, it can be inferred that the addition of an enzyme improved and accelerated the performance of the detergent solution.

## Conclusion

The properties of the protease of *A. terreus* were investigated. The enzyme was a thermostable alkaline serine protease and showed high affinity for various protein substrates, stability and compatibility when mixed with surfactants, bleaches, oxidizing agents and local powder detergents. In addition, it was able to completely destain blood on white cloth. These properties suggested the present enzyme to be commercially exploited as an ideal candidate for detergent preparations.
